# Response of diademed sifaka (*Propithecus diadema*) to fosa (*Cryptoprocta ferox*) predation in the Betampona Strict Nature Reserve, Madagascar

**DOI:** 10.1002/ece3.11248

**Published:** 2024-04-09

**Authors:** G. Bonadonna, O. M. Ramilijaona, B. M. Raharivololona, A. Andrianarimisa, H. Razafindraibe, K. Freeman, F. Rasambainarivo, E. E. Wroblewski, K. M. Milich

**Affiliations:** ^1^ Department of Anthropology Washington University in St. Louis St. Louis Missouri USA; ^2^ Zoology and Animal Biodiversity, Faculty of Sciences University of Antananarivo Antananarivo Madagascar; ^3^ Department of Anthropology and Sustainable Development, Faculty of Sciences University of Antananarivo Antananarivo Madagascar; ^4^ Madagascar Fauna and Flora Group Kalinka UK; ^5^ Department of Biology East Carolina University Greenville North Carolina USA; ^6^ Mahaliana Labs SARL Antananarivo Madagascar

**Keywords:** conservation, *Cryptoprocta ferox*, fragmented habitat, isolated habitat, lemur anti‐predator strategy, predator–prey dynamics, *Propithecus diadema*

## Abstract

Large‐bodied mammals living in fragmented habitats are at higher risk of extinction, and such risk can be influenced by ecological factors such as predator–prey system dynamics. These dynamics can be particularly complex for conservation management when one endangered species preys on another endangered species in an isolated or poor‐quality habitat. Here we describe predation events observed over 19 months that involved two threatened species: the largest carnivore in Madagascar, the fosa (*Cryptoprocta ferox*), and three groups of diademed sifaka (*Propithecus diadema*) in the Betampona Strict Nature Reserve. This site is a 22 km^2^ low‐altitude rainforest that is surrounded by agricultural land and isolated from larger forest corridors. We aim to (1) assess the behavioral changes of *P. diadema* in response to fosa attacks and identify any antipredator strategies that they adopted, and (2) quantify the frequency of fosa attacks and the predation impact on the sifaka population. We report five direct observations of fosa predation attempts (one successful), the discovery of a dead sifaka with evidence of fosa predation, and the disappearance of three individuals. We describe the observed attacks and compare the sifaka activity budgets and movement patterns before and after the events. To escape the predator, sifakas fled short distances, hid, and remained vigilant. The impact of predation, combined with low reproductive rates and potentially high inbreeding of this isolated diademed sifaka population, could affect the survival of this species in Betampona. Given the compounding effects of habitat isolation and high hunting pressure, community‐specific conservation strategies should incorporate predator–prey dynamics via longitudinal monitoring of predator and prey population densities and quantifying the predation pressure between them.

## Graphical abstract

Our study explores predator‐prey dynamics in a fragmented habitat, focusing on the consequences of fosa predation for three groups of diademed sifakas over 19 months of observation in the Betampona Strict Nature Reserve, Madagascar. We report five direct observations of fosa predation attempts, of which one was successful, the discovery of the body of a sifaka with evidence of fosa predation, and the disappearance of three individuals. The sifakas' strategy to escape fosa predation was to flee short distances, hide, and remain vigilant. This research offers insights into how predator‐prey ecology affects the conservation of threatened species and their habitat.

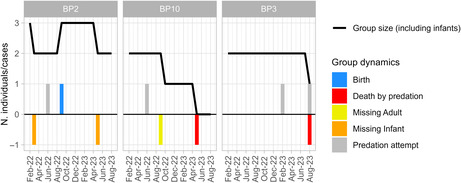

## 1 INTRODUCTION

Pervasive anthropogenic habitat modification directly impacts endangered species, but the resulting implications for predator–prey dynamics are not well understood (Dorresteijn et al., 2015). These dynamics can be particularly complex for conservation management when one endangered species is known to prey on another endangered species within the confines of isolated or poor‐quality habitat. Large‐bodied mammals living in fragmented habitat are at higher risk of extinction (Crooks et al., 2017), and such risk can be influenced by ecological factors such as predator–prey system dynamics (Karsai & Kampis, 2011).

In predator–prey systems, prey and predator abundance, as well as habitat carrying capacity and environmental fluctuations, can affect population densities and growth (Abrams, 2000; Arditi & Ginzburg, 1989; Bandyopadhyay & Chattopadhyay, 2005). Additionally, the feeding strategy of predators (e.g., one or many prey species) and the restriction of habitat use for both prey and predators (e.g., more or less limited to specific habitats) influence the effects that predators have on prey populations and vice versa (Ryall & Fahrig, 2006). In Madagascar, terrestrial apex predators and primates tend to co‐occur and be restricted to forested habitats (Farris et al., 2014; Gerber et al., 2012b).

Lemurs have several predator species native to Madagascar, including 10 raptors, seven carnivorans, and six reptiles (Fichtel & Kappeler, 2002; Goodman, 2003; Goodman et al., 1993; Hart, 2007; Karpanty & Goodman, 1999; Karpanty & Wright, 2007; Sauther, 1989; Wright, 1998). Other non‐native predators are domestic dogs (*Canis familiaris*) and wild or feral cats (*Felis* spp.) (Farris et al., 2014; Gerber et al., 2012b; Goodman, 2003; Hart, 2007). Over 90% of reported predation events on lemurs are attributable to raptors and carnivorans (Hart, 2007), and of those events, over 75% were on lemurs weighing less than 2 kg, with the genera *Microcebus* and *Cheirogaleus* at highest predation risk (Hart, 2007; Scheumann et al., 2007). However, raptors were observed to prey on bigger diurnal lemurs, including *Varecia* and *Propithecus* species, in addition to smaller nocturnal lemurs, removing 1%–21% of the lemur population (Brockman, 2003; Karpanty, 2006; Karpanty & Wright, 2007).

The fosa (*Cryptoprocta ferox*) is the biggest carnivore in Madagascar, with a body mass of 5–9 kg (Gerber & Hawkins, 2022; Hawkins, 1998; Lührs & Kappeler, 2014). Fosas are agile and effective predators; their diet includes birds, reptiles, amphibians, and insects, although they prey preferentially on mammals and have a high percentage of primates in their diet (Hawkins & Racey, 2008), including large‐bodied lemurs that can be more than half of their body mass (Dollar et al., 2007; Hawkins & Racey, 2008; Wright et al., 1997). Fosa scat has contained evidence of terrestrial and arboreal lemur species, as well as diurnal, crepuscular, and nocturnal species, suggesting a flexible hunting strategy (Dollar et al., 2007; Farris et al., 2015; Goodman et al., 1993; Hawkins & Racey, 2008; Wright et al., 1997). Fosas also eavesdrop on prey alarm calls to locate them (Fichtel, 2009).

The fosa is categorized as Vulnerable by the IUCN (2022). Therefore, the fosa is at risk of extinction as are almost all of its lemur prey (IUCN, 2022), which makes understanding the dynamics of this predator–prey system critical to the conservation of both wild lemur and fosa populations. Fosa and the other smaller endemic carnivorans species of Madagascar are also susceptible to habitat fragmentation, anthropogenic disturbance, and the presence of domestic animals or exotic carnivores in the forests where they range, although selective logging does not seem to affect their density (Farris et al., 2014, 2017; Gerber et al., 2010, 2012a, 2012b; Merson et al., 2020). Direct observations of fosa predation attempts are rare, making such observations important to document. To our knowledge, there is only one reported observation of a lemur predation attempt by a fosa, which was on an aye‐aye in Torotorofotsy (Sefczek et al., 2018). Most information about fosa‐lemur dynamics comes from lemur corpses with evidence of fosa predation and analysis of fosa scat content (Dollar et al., 2007; Hawkins & Racey, 2008; Irwin et al., 2009; Karpanty & Wright, 2007; Wright et al., 1997).

As a result of intense predation risk, lemurs evolved several anti‐predator strategies shaped by predator hunting style and lemur body mass (Isbell, 1994; Scheumann et al., 2007). Anti‐predator behavioral adaptations include cathemerality, increasing group size or concealment, selecting safe sleeping sites, forming small sleeping parties, and increasing vigilance (Colquhoun, 2006; Curtis, 2007; Gould & Sauther, 2007; Janson & Goldsmith, 1995; Rasmussen, 2005; Wright, 1998). Lemur responses to predators include emitting panic or predator‐specific alarm calls, mobbing, hiding, and escaping (Karpanty & Wright, 2007; Scheumann et al., 2007; Stanford, 2002). Lemurs also recognize and react to heterospecific alarm calls (Fichtel, 2004; Fichtel & Kappeler, 2002; Magrath et al., 2015; Scheumann et al., 2007).

Diademed sifakas (*Propithecus diadema*) are among the largest lemurs with an average weight of 6.5 kg (Gordon et al., 2013; Powzyk & Mowry, 2003). The species is classified as Critically Endangered (IUCN Red List, 2022) with the major threats being habitat loss and unsustainable hunting pressure (Borgerson et al., 2021; IUCN Red List, 2022). These diurnal foli‐frugivore lemurs live in Eastern Madagascar in multimale‐multifemale groups composed of two to eight individuals (Irwin, 2008a, 2008b; Powzyk, 1997; Weir, 2014), give birth between May and August, and wean infants at 25 weeks of age (Weir, 2014). This species emits contact calls and distinct alarm calls in response to terrestrial and aerial predators (Fichtel & Kappeler, 2002; Petter & Charles‐Dominique, 1979; Valente et al., 2022). Fosas are likely the only carnivore to prey on adult sifakas (Patel, 2005; Wright, 1998), given the size of both species, and fosas affected population densities and caused group extinction in Tsinjoarivo forest fragments and Ranomafana National Park (Irwin et al., 2009; Wright et al., 2012). Fosas have seasonal peaks of predation activity between April and October (Irwin et al., 2009; Karpanty & Wright, 2007), which is considered as the lean dry season and when the sifaka rely more on leaves for their diet (Irwin, 2008b). This peak predation period also coincides with some or all of the gestation, birth, and lactation periods of diademed sifakas.

Both diademed sifakas and fosas live in the Betampona Strict Nature Reserve (hereafter Betampona), a 2228 ha low‐altitude rainforest fragment surrounded by agricultural land that is approximately at a 20–25 km linear distance from the closest habitat of the Ankeniheny‐Zahamena forest corridor (CAZ). The diademed sifaka population at Betampona is small (estimated at 16 individuals prior to the beginning of the study and at 13 individuals in eight groups at the end of 2022), isolated, and having low reproductive success, with 10 births recorded among four groups and 70% of infants and juveniles not surviving between 2018 and 2022 (MFG annual report 2019, 2020–2022). The fosa population in the reserve is the subject of an ongoing study of their health in which individuals are monitored and some are collared (Rasambainarivo et al., 2018). Betampona's isolation prevents natural migration for lemurs and, most likely, carnivorans like fosas, which select forested habitat and avoid ranging in agricultural land (Wyza et al., 2020). Given the correlation between high extinction risk and body mass (Turvey & Fritz, 2011) and the time‐lagged effects of habitat fragmentation to which carnivorans and primates in Madagascar are particularly vulnerable (Broekman et al., 2022), diademed sifaka and fosa are species at high risk of local extinction in the isolated forest of Betampona. Thus, Betampona is a critical site for understanding the predator–prey dynamics between these species.

Here we describe five direct observations of fosa predation attempts, of which one was successful, the discovery of the body of a sifaka with evidence of fosa predation, and the disappearance of three individuals that occurred over a 19‐month study period for three neighboring groups of diademed sifakas in Betampona. We describe the attacks and compare the sifaka activity budgets and movement patterns before and after the events when possible. Given that direct observations of fosa predation on lemurs are rare, these observations allow us to address two primary research questions: (1) what are the behavioral responses of *P. diadema* to fosa attacks (i.e., what are *P. diadema* antipredator strategies), and (2) what is the frequency of fosa attacks and the predation impact on an isolated population of *P.diadema* in a forest fragment? We also discuss the conservation implications for diademed sifakas at Betampona.

## 2 METHODS

### 2.1 Study site and subjects

**FIGURE 1:**
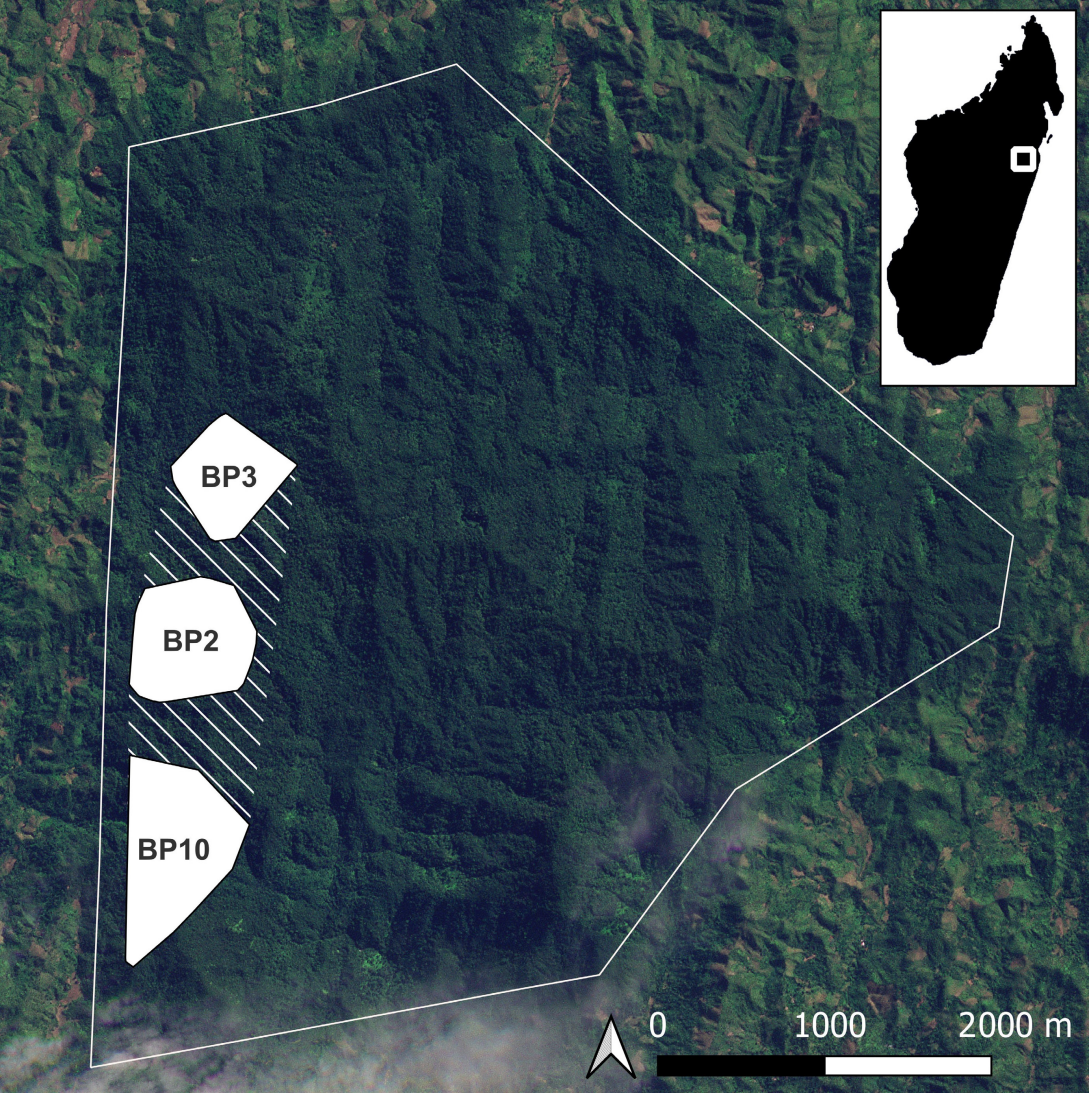
Location and boundaries of the study site and diademed sifaka group ranges. The boundary of the Betampona Strict Nature Reserve is marked by a solid white line. The white‐filled polygons within the reserve indicate the home ranges (Minimum Convex Polygons) of the three diademed sifaka study groups (BP2, BP3, BP10), each having one or two direct observations of fosa attacks. The total area of the three home ranges, together with the dashed area that connects them, is 235.6 ha (satellite image from ©Planet Labs PBC (www.planet.com)).

**FIGURE 2:**
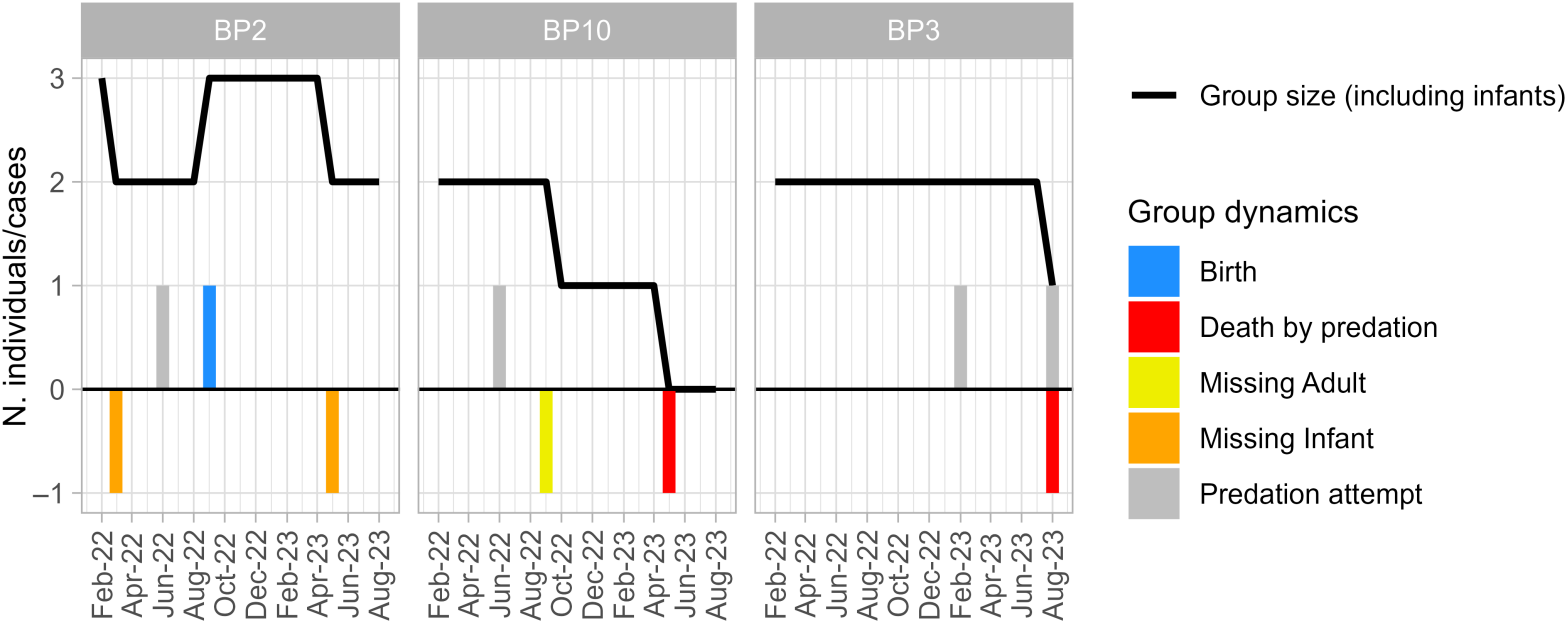
Monthly diademed sifaka group sizes (line) and cases of dynamics (barplot) during the study period (19 months).

Betampona Strict Nature Reserve (49°12′ E, 17°55′ S), located 40 km northwest of Toamasina, is one of the last fragments of lowland rainforest once present along the coast of eastern Madagascar. It is managed by Madagascar National Parks with the NGO Madagascar Fauna and Flora Group (MFG) as a conservation and research partner. The 22.28 km^2^ reserve is surrounded by human‐modified landscapes and became isolated through extensive deforestation (Figure 1). Nevertheless, it is a biodiversity‐rich site harboring 27 endemic mammal species, which include five carnivorans (including fosa) and 11 lemur species (Freeman et al., 2014; Golden et al., 2014). We report data collected on three groups of diademed sifaka (BP2, BP3, BP10) between February 2022 and August 2023 (19 months). At the onset of the study, each group was composed of two adults: a male and a female (see Figure 2 for group composition dynamics over the study period). At least one adult individual in each group was fitted with a radio collar (ATS, M2930) and a tag to facilitate tracking and identification. All individuals were also identified by their unique markings.

### 2.2 Data collection

We conducted individual dawn‐to‐dusk focal follows (Altmann, 1974) of all adults in each group, following one individual per day, five days per week, rotating among individuals daily and among the three groups weekly. It was not always possible to visually track all adults because they do not all have radio collars. The distance from the field station to groups BP2 and BP3 required researchers to camp during the weeks that those groups were followed and resulted in focal follows starting later on the first day of the week after a camp is established and a group is located. The research was typically conducted in teams of two, but there were three people present during the predation attack on the BP10 group (described below).

During daily focal follows, we recorded the following behaviors at 5‐min‐interval instantaneous scans: resting, vigilance, traveling, feeding, self‐grooming, social interactions, not visible (i.e., present but out of sight), and “other” for behaviors not listed. We recorded the focal as not available when we did not know the location of the focal individual, which is different from the "not visible" category described above. For each scan, we visually estimated the following parameters: the categorical location of the focal individual with respect to the canopy (in the upper, middle, or lower canopy of a tree, on the trunk, or on the ground), the tree's diameter at breast height (DBH) (up to 20, 21–40, 41–60, 61–80, 81–100 cm, more than 100 cm), the tree's height from the ground (up to 10, 11–15, 16–0, 21–25 m, more than 25 m), and the distance of the focal from the other member of the group (contact, <1, 1–5, >5 and ≤10 m, >10 m, or undetectable) within a 25 m radius. We also recorded the duration of each feeding bout and all occurrences of unidirectional or bidirectional allogrooming, agonistic interactions, scent marking, and vocalizations (“Zzuss,” “Zzuss‐tsk,” and “Roar” alarm calls, “Howl” lost contact and cohesion call, and “Hum” lost contact and disturbance call as defined by Patel & Owren, 2012 and Valente et al., 2022).

We collected the spatial coordinates of the focal individual at 15‐minute intervals by using a handheld GPS unit (GARMIN GPSMAP 65s). A waypoint was recorded with an accuracy of 5 m, and the observers maintained a 5–15 m distance from the focal animal. If the focal was lost (i.e., not available) for more than 15 min, we recorded the first position available once the focal was found. We also recorded all births and deaths or missing individuals within the study period. Because the target of our observations was the diademed sifakas, we did not have a specific protocol to record the fosa behavior, but we opportunistically recorded and described as many details as possible during the predation events that took place.

### 2.3 Data analysis

We report spatial and behavioral data collected on three diademed sifaka groups in the context of four unsuccessful fosa predation attempts, one each for BP2 and B10, and two for BP3 (approximately 6 months apart – February and August 2023). An additional, successful fosa attack on BP3 was observed in August 2023, in which a female was killed. This event was observed eight days prior to the unsuccessful attempt on the remaining individual in that group, but it was not observed during data collection by the research team, and, therefore, less detail is available for this event. We include the details available for this successful attack (Appendix S1); however, the four attempts for which more data are available are the focus of our analyses.

To describe the immediate response of the diademed sifakas to the fosas' predation attempts, we present the time series of the behaviors displayed by the focal sifakas on the day of the attack. For statistical analyses, we used behavioral data after the attack for the following three phases: the 5‐min scans after the attack on the day of the predation attempt (T0), the remainder of the week after the day of the predation attempt (T1), and the first week of data for a group after the week of the attack, which corresponded to 19–21 days later (T2). We compared behavior after the attacks with daily behavioral frequencies before the attack (PRE) collected during the first week available before each attack (between 16 and 36 days before attacks), the days immediately before each attack when available, and the day of the attack up to the predation attempt. We excluded data collected on August 15 and August 16, 2023, preceding the attack on BP3 from the PRE dataset because the female of group BP3 was killed by a fosa the week before the attack on August 16, 2023, potentially impacting the behavior of the remaining individual in the group and, therefore, not truly representing a “PRE” condition. We calculated daily frequencies for each behavior as proportion of the total number of scans recorded in a day, excluding scans when the focal was not detectable (i.e., not available) by the observer (NA) (Table S1).

We tested whether there was an effect of the predation attempts on the diademed sifaka behaviors by fitting linear mixed effect models (function lmer) using the package lme4 (Bates et al., 2015) in R 4.2.2 (R Core Team, 2022). We fit models for the dependent variable of daily proportion of 5‐min scans for each behavior; we included the four periods relative to the attacks as fixed effect (PRE, T0, T1, T2), and the sifaka groups for each attack as random effect to account for the multiple daily observations on each group before and after the four attacks. We checked for normality by visually checking q‐q plots of model residuals for each behavior. We assessed the model using a type III ANOVA to generate F‐stats and *p*‐values. In case of significant effects, we applied Tukey's post hoc tests to compare mean contrasts between the different factor levels (PRE, T0, T1, T2) using the “emmeans” function from the emmeans package (Lenth, 2019).

Daily path length (DPL) calculations include segments for which we could not directly see the individuals traveling between points, and the sampling interval is more than 15 min. To avoid the bias of correlation between observation hours and DPLs in the statistical analyses, we calculated the rate meters per hour (DPL m/h) obtained by dividing the DPL by the number of observation hours for each day (Table S1). We fit a linear mixed model (lmer function), including the groups as random effect, followed by a type III ANOVA to test the effect of the attack phases on the DPL. We log‐transformed the DPL m/h to have a normal distribution of model residuals, visually checked with q‐q plots. We used the software QGIS Development Team (2022) version 3.24 for all spatial analyses. We used R software (R Core Team, 2022; version 4.2.2) for all statistical analyses.

In addition to the predation events, we report all cases of sifaka births, disappearances, and deaths that occurred between February 2022 and August 2023 (Figure 2). We calculated the group annual off‐take rate following Irwin et al. (2009). We first calculated the group‐years by multiplying the number of groups (three) by the study period in years (19 months equivalent to 1.6 years), obtaining 4.8 group‐years. We then divided the number of fatalities by the group‐years.

### 2.4 Ethical note

This research was approved by the Washington University in St. Louis Institutional Animal Care and Use Committee (IACUC # 19‐0962), the Malagasy Ministry of Environment and Sustainable Development, and Madagascar National Parks (permits N. 134 and N. 333/22/MEDD/SG/DGGE/DAPRNE/SCBE.Re). Data collection methods conformed to the national legislation and international regulations concerning animal welfare.

## 3 RESULTS

The sifaka groups neighbor one another, and their home ranges together cover an area of 235.6 ha (including the space between group ranges) (Figure 1). Their home ranges are aligned north–south along the southwestern border of Betampona, BP3 in the north, BP10 in the south, and BP2 between them.

### 3.1 *P. diadema* anti‐predator strategies

We collected data on four fosa predation attempts on three diademed sifaka groups (Figure 2). The first two attacks occurred one week apart: the first attack was on the adult female of BP2 on 1 June 2022 at 8:20 AM, and the second was on the male of BP10 on 8 June 2022 at 12:17 PM. In both cases, a single fosa attacked the lemurs, but we could not determine if the two attacks were by the same animal. In the third attack, two fosas – of which one was collared – attacked the two individuals of BP3 on 27 February 2023 at 5:00 PM. The fourth attack, also observed on BP3, took place on 16 August 2023 at 1:33 PM and involved a single male sifaka and a single fosa. This case happened eight days after BP3 was seen being chased by a fosa, and the female was eaten by the predator, a fifth attack for which we do not have systematic data but is described in Appendix S1. Detailed descriptions of the attacks are in Appendix S1. To briefly summarize the attacks, as soon as sifakas perceived the presence of a fosa, they started a 3–10 min sequence of “Zzuss” and “Zzuss‐tsk,” their specific alarm call for terrestrial predators (Figure S1). In all cases, fosas did not pursue their prey after an initial chase, once the sifaka reached a certain distance and/or stopped vocalizing. Also, the fosas completely ignored the human presence, walking as close as −1.5 ‐ 2 m from the researchers.

In total, we analyzed behavioral and ranging data collected during 384.67 h of observation (4529 scans) across 46 days in relation to the attacks. The baseline pre‐attack data (PRE) consist of 149 hours (1788 scans) across 21 days, including the days of the attacks; same‐day post‐attack data (T0) include 14.92 h of observation (179 scans) over 4 days (one attack for each day); data after the attacks during the week of the predation attempts (T1) sum to 71.67 h (860 scans) over 8 days; data collected during the week following the predation attempts (T2) include 128.75 h (1545 scans) across 16 days (Table S1).

**FIGURE 3:**
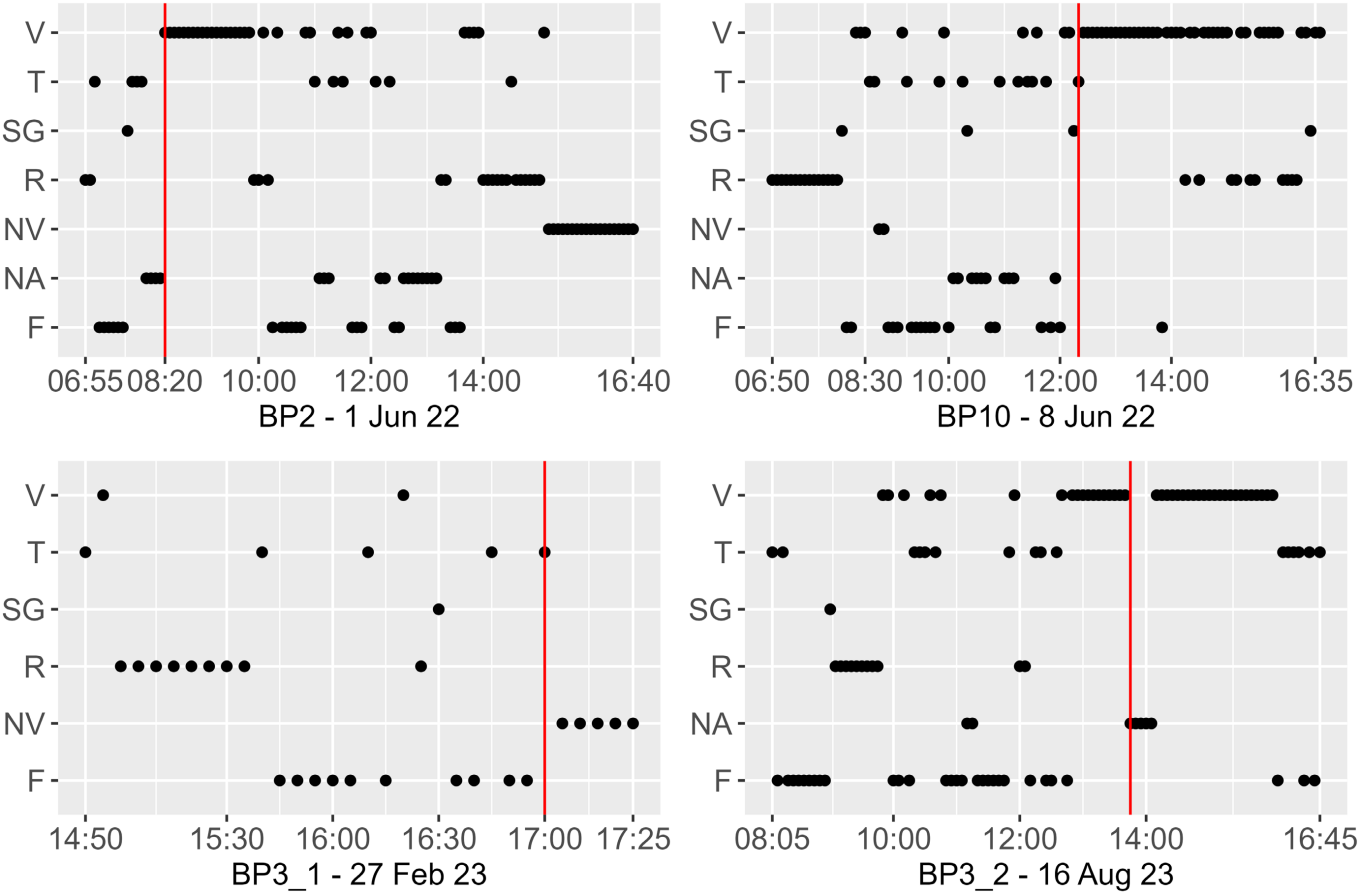
Chronological sequence of the behavior displayed by the focal individuals at each 5‐min instantaneous scan on the day of the attack for each group, the red solid lines represent the time of the attacks. F, feeding; NA, not available/focal lost; NV, non‐visible; R, resting; SG, self‐grooming; SOC, social interactions; T, traveling; V, vigilance.

**FIGURE 4:**
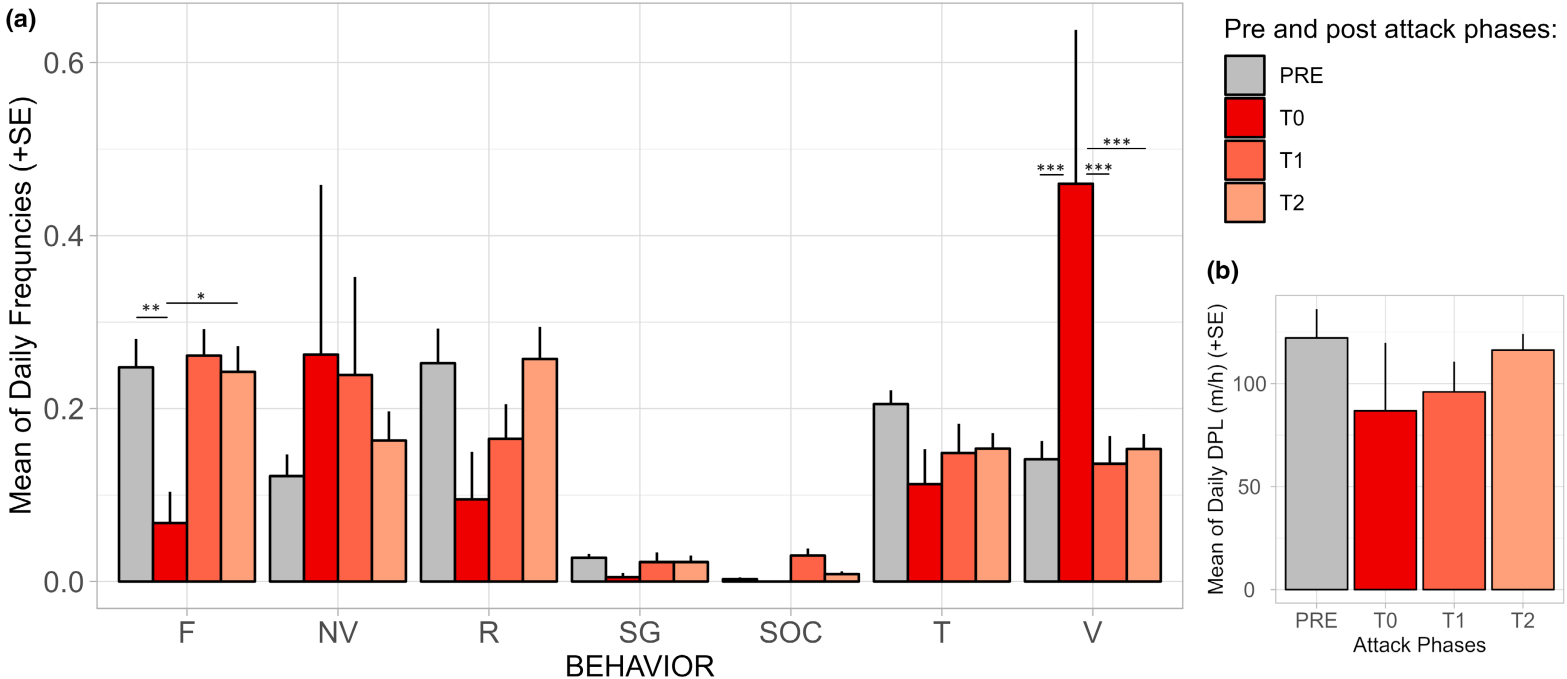
Average of daily behavioral frequencies (a) and daily path length in meters per hour (DPL m/h) (b) for each phase before or after the four fosa attacks: before (PRE), same day after the attack (T0), day after the attack (T1), first week of observation on each group after the attack (T2). Error bars represent the standard error (SE). Different letters indicate post hoc Tukey's HSD test significant differences between phases for a given behavior (.001 “***”; .01 “**”; .05 “*”). F, feeding; NV, non‐visible; R, resting; SG, self‐grooming; SOC, social interactions; T, traveling; V, vigilance.

Immediately after the attack, there was a change in the behavioral pattern of the diademed sifakas, with individuals being almost exclusively vigilant during the first couple of hours after the attacks (Figure 3). Vigilance increased significantly after fosa attacks (lmer *F*_3,43_ = 8.497, *p* < .001, Table 1), and feeding (lmer *F*_3,42.4_ = 3.623, *p* = .02, Table 1) significantly decreased after attacks; traveling decreased after the attacks although it was at the limit of being significant (lmer *F*_3,43.5_ = 2.678, *p* = .059, Table 1) (Figure 4a). We did not find a significant effect of the attacks on the frequency of resting (lmer *F*_3,45_ = 1.716, *p* = .177, Table 1), self‐grooming (lmer *F*_3,45_ = 0.899, *p* = .45, Table 1), nor on the proportion of scans during which the diademed sifakas were non‐visible (lmer *F*_3,45_ = 1.052, *p* = .379, Table 1), despite non‐visible scans being more frequent on the day of and the day after the predation attempt (Figure 4a). Social interactions were not observed immediately after the attack (T0) and were more frequent the day after the attack (Figure 4a), but we excluded social interactions from statistical tests because residuals were not normally distributed due to the high frequency of zero as this behavior was rarely observed (Table S1). The post hoc Tukey pairwise analysis showed that behavioral frequencies were mostly affected immediately after the attack (T0) compared to the other two post‐attack phases (Figure 4a, Table S2). Vigilance was significantly higher during T0 compared to any other phase (Figure 4a, Table S2), feeding was significantly lower in T0 than PRE and T2 (Figure 4a, Table S2). We did not detect a difference in behavioral frequencies between pre‐attack data (PRE) and T2 (Table S2).

**TABLE 1:** Summary of linear mixed‐effects model for each behavior and DPL m/h.

Behavior	Fixed factor	Estimate *β*	CI 95%	SE	df	*t*‐Value
**Feeding**	T0	−.189	−0.300, −0.077	0.058	42.27	−3.27
	T1	−.024	−0.109, 0.065	0.045	42.57	−0.53
	T2	−.016	−0.084, 0.052	0.035	42.25	−0.46
**Traveling**	T0	−.093	−0.173, −0.013	0.042	42.75	−2.22
	T1	−.057	−0.117, 0.004	0.032	44.81	−1.78
	T2	−.052	−0.100, −0.003	0.025	43.06	−2.03
**Vigilance**	T0	.320	0.194, 0.446	0.065	42.65	4.92
	T1	.007	−0.094, 0.102	0.050	43.61	0.13
	T2	.017	−0.061, 0.093	0.040	42.68	0.44
Resting	T0	−.157	−0.323, 0.008	0.086	45.00	−1.82
	T1	−.087	−0.213, 0.039	0.066	45.00	−1.33
	T2	.005	−0.095, 0.106	0.053	45.00	0.10
Self‐grooming	T0	−.023	−0.049, 0.004	0.014	45.00	−1.63
	T1	−.005	−0.025, 0.015	0.011	45.00	−0.48
	T2	−.005	−0.021, 0.011	0.008	45.00	−0.61
Non‐visible	T0	.141	−0.064, 0.345	0.107	45.00	1.32
	T1	.117	−0.039, 0.272	0.081	45.00	1.44
	T2	.041	−0.083, 0.165	0.065	45.00	0.64
DPL (m/h)	T0	−35.488	−90, 19	28.327	45.00	−1.253
	T1	−26.238	−67, 15	21.573	45.00	−1.216
	T2	−5.926	−39, 27	17.231	45.00	−0.344

*Note*: The predictor is the daily frequency for each behavior or the DPL, the fixed effect is the pre‐ and post‐attack phases (T0, T1, T2) with results relative to before the attacks, and diademed sifaka groups are the random effect (we coded group BP3 differently for each predation attempt case, total number of groups = 4). Behaviors in bold are those significantly affected by fosa attacks.

**FIGURE 5:**
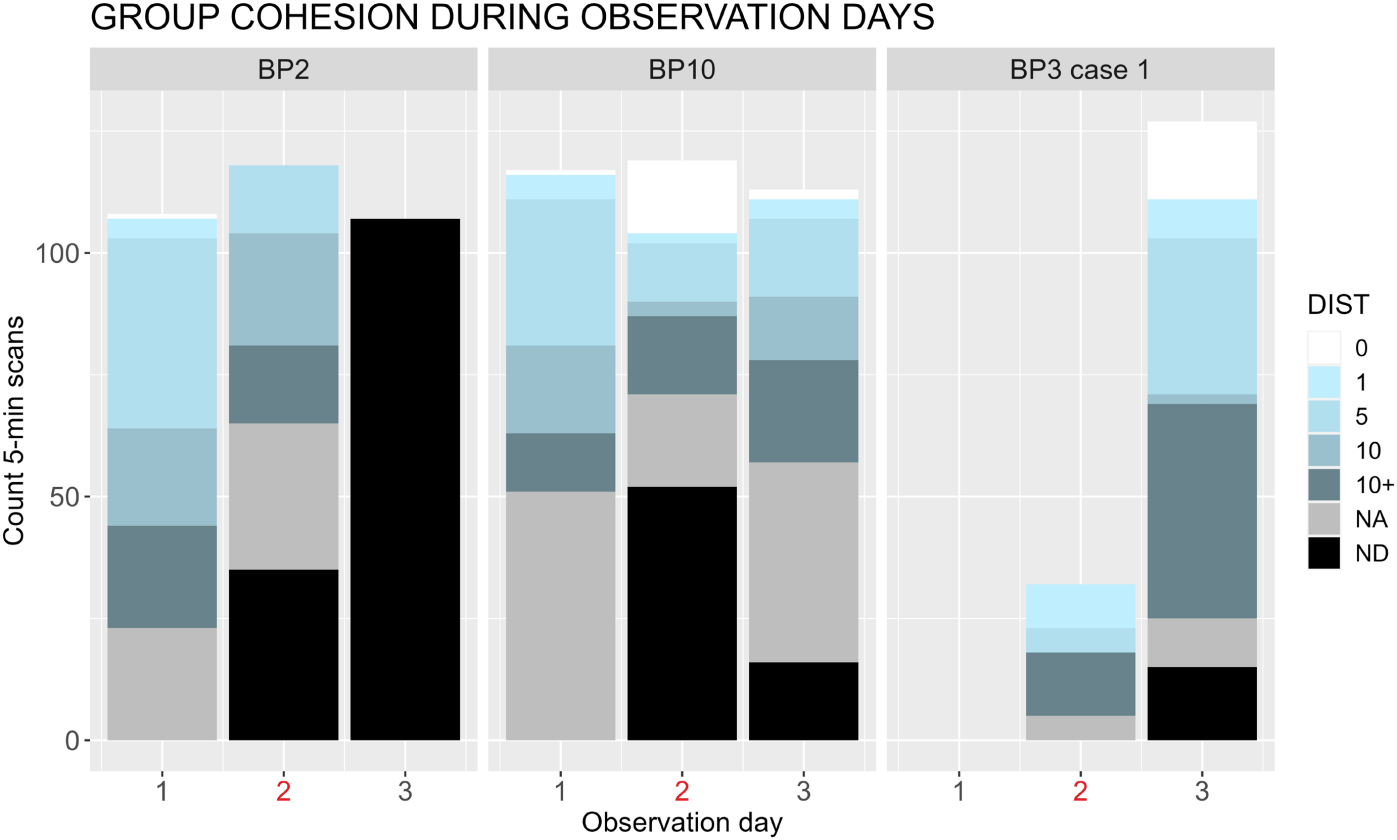
Group cohesion measured as the categorical distance in meters (DIST) between individuals at each 5‐min instantaneous scan during the day before (1), the day of (2, in red), and the day after the attack (3). ND: no other conspecific presence detectable within a 25 m radius of the focal individual, NA: the focal was lost or not available. Time of the attack: BP2, 8:20 AM; BP10, 12:17 PM; BP3, 5:00 PM. Only the first case reported for BP3 is included in the graph as in the second case there was only one individual in the group.

From a descriptive analysis of the distance between individuals in each group on the day before, the day of, and the day after the attack, we found that the sifaka groups became less cohesive after attacks. The sifaka pairs of BP10 and BP3 split after the attack and did not rejoin until the following day (Figure 5). In BP2, the focal spent more time alone after the attack, and only one individual was detected the day after the attack (Figure 5).

**FIGURE 6:**
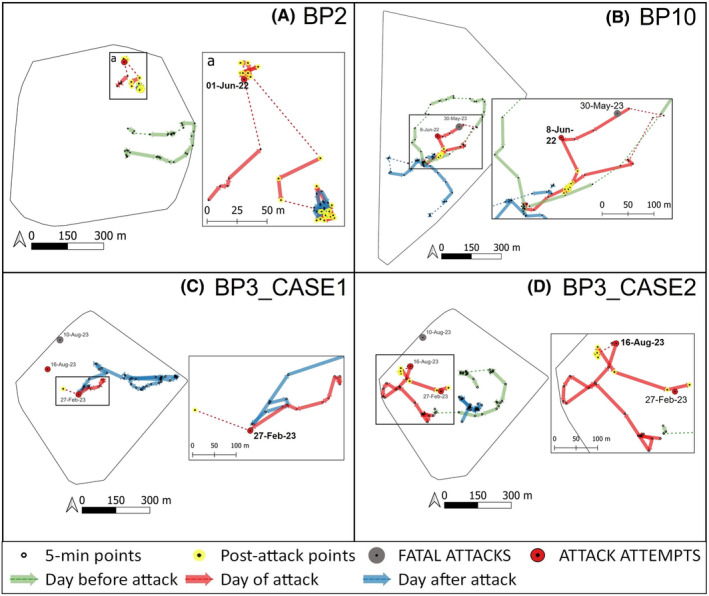
Maps of the home range of each group (A–D) showing the daily paths of the day before, of, and after the attacks (the dashed lines indicate segments for which we could not see the individuals traveling between points, and the sampling interval is over 15 min). The 3–15 m shifting range of the GPS waypoints when the individual was stationary is due to small shifts of the person holding the GPS trying to get a better sight of the focal and/or a change in satellite reception. Red dots mark the attack locations, and yellow dots are post‐attack locations recorded on the same day. The gray dots mark where sifaka bodies were found following the death from fosa predation.

After the predation attempts, the sifakas did not move very far from the attack location (Figure 6), and on average, the DPL adjusted for the observation time (meters per hour, DPL m/h) was shortest for T0 (Figure 4b). However, we did not find a significant effect of the attacks on the DPL (*F*_3,45_ = 1.8698, *p* = .464). These results need to be considered carefully given the wide confidence intervals for T0 (Table 1).

In summary, the immediate response of diademed sifakas consisted of alarm calling and altering cohesion and ranging patterns, indicating a strategy of alerting followed by fleeing short distances, hiding, and remaining vigilant to escape the predators. Behavioral frequencies were comparable to the pre‐attack phase in the days immediately following the attack and by the time we rotated back to the individuals after the attack, indicating a short‐term behavioral alteration.

### 3.2 Fosa predation rates on *P. diadema*

**FIGURE 7:**
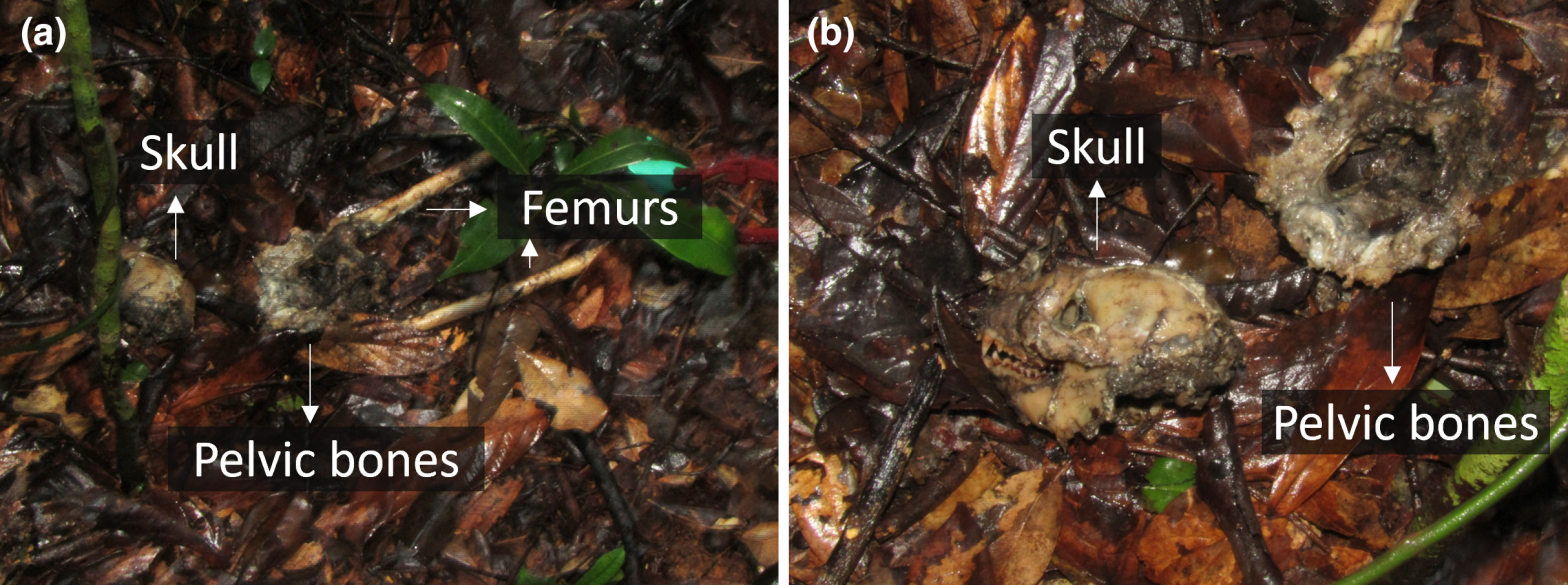
Wider (a) and closer (b) view pictures of the remains of the male of group BP10 showing the skull, pelvic bones, and femurs. © O.M. Ramilijaona.

Within the same time frame as the observed predation attempts, we recorded the disappearance of three individuals (two infants from BP2 when they were 5 and 7 months old, and an adult female for BP10) (Figure 2) for which fosa predation cannot be ruled out and the death of one individual (a male in BP10) who was preyed upon by a fosa. Given that infant sifakas as young as 5 and 7 months old cannot survive independently, we can assume that these individuals died. Also, given the small population size in Betampona (16 individuals at the time of this study) and that all individuals are monitored for demographic changes, we could confirm that the female had not transferred to a different group. We were also unable to detect a signal from her radio collar, further suggesting that she had not migrated within the population. Therefore, she was also presumed dead. On 30 May 2023, we found the remains of the single BP10 male who had likely been preyed upon by one or more fosas (Figure 2). This male had been part of the unsuccessful fosa attack on BP10 in June 2022 along with the female who later disappeared, potentially as a result of predation, in September 2022. The male's body was found in an area that had been used by the individual as a sleeping site, 117 m from the fosa attack location reported in June 2022 (Figure 6B). The nylon radio collar with marks on it (most likely from teeth) was next to the corpse, which consisted of the skull, the pelvic bones, and femurs with hairy skin around it (Figure 7). We also found hairs of the individual scattered throughout the area and the abdominal contents about 5 m from the collar and the bones. The death of the male eliminated the group BP10. In the three months following the end of group BP10 that we were conducting fieldwork, there were no signs that other diademed sifakas began using BP10's home range.

If we consider the two confirmed kills, the female that disappeared from group BP10, and the two infants that disappeared before dispersal age in areas with fosa activity in the three groups over 19 months (1.6 years), we obtain a total of five fosa killings, which represents an average annual offtake rate of 1.04 individuals per group (five events over 4.8 group‐years) (Figure 2). If we only consider the two confirmed kills, we obtain an average annual offtake rate of 0.42 per group.

## 4 DISCUSSION

Predation is a major selective force that influences the behavioral ecology of prey species (Cheney & Wrangham, 1987; Dawkins & Krebs, 1979; Hill & Dunbar, 1998; Isbell, 1994). Understanding predator–prey dynamics and anti‐predator behaviors of endangered prey species is critical for conservation management and restoration efforts. Here, we report a detailed description of four unsuccessful predation attempts and two successful predation events by *C. ferox* on both male and female *P. diadema* belonging to three neighboring groups in the Betampona Strict Nature Reserve. We also report disappearances in two of the groups that could be related to fosa predation, although we cannot exclude other causes such as hunting by humans (especially for the adult female in BP10 as the radio collar was not detected after the disappearance) or dogs as they are frequently detected within the reserve (Rasambainarivo et al., 2017). Based on behavioral frequencies, all groups reduced their feeding, traveling, and resting and increased their vigilance after the attacks. The individuals stayed apart or reduced their detectability because, after the attacks, we could only assess the presence of the focal animal, and individuals were alone on the day of, and on the day after the attacks.

Most predation events of lemurs by fosa are inferred from remains found after the fact. Our observations of both unsuccessful and successful attacks are a rare opportunity to evaluate the behavior and strategies of both the fosa and the sifaka. All the predation attempts that we observed were consistent with the ambush hunting strategy adopted by this carnivore (Irwin et al., 2009; Wright et al., 1997) as the fosas did not pursue their prey after an initial chase. The costs of chasing lemurs in the canopy likely outweigh the probability of a successful hunt, especially after fosas lose the element of surprise, and the lemurs are alerted to employ their anti‐predator behaviors. We observed both sifakas in the groups emitting alarm calls upon detection of the predator, during, and immediately after the predation attempts while fleeing the predator, and even the sole remaining individual of BP2 emitted Zzuss‐tsk during and after the predation attempt. The sifakas stopped alarming shortly after escaping the attack. Wright (1998) suggested that the Zzuss vocalization, emitted prior to escaping, served a mobbing function against fosas as a form of threat towards the predator. However, in our case, the sifakas did not approach or harass the fosas while emitting Zzuss and Zzuss‐tsk, as in mobbing responses described for other primates (reviewed in Crofoot, 2013). The Zzuss and Zzuss‐tsk appeared to serve as an alert for conspecifics which, potentially, disrupts the surprise effect of the predator and deters its continued pursuit of the prey (Woodland et al., 1980). The “pursuit deterrent” function (Woodland et al., 1980) has been suggested for alarm calls against predators with an ambush hunting strategy as given by red‐tailed sportive lemurs (*Lepilemur ruficaudatus*) (Fichtel, 2007), and monkey species of the genera *Pithecia*, *Colobus*, *Cercopithecus*, and *Cercocebus* against leopards and jaguars (Adams & Kitchen, 2020; Schel et al., 2010; Zuberbühler et al., 1999).

A common anti‐predator escape response strategy of primates after a predator is detected is to flee and conceal (Isbell, 1994; Stanford, 2002). Fleeing results in longer travel distances following an encounter with a predator than before the encounter, as seen in red colobus monkeys (*Colobus badius*) (Gebo et al., 1994). However, we observed that the sifakas reduced their travel distance following the predation attempts. Freezing and concealing can reduce detectability especially in the case of small, nocturnal species, or species grouping in small parties, as reported in the gray mouse lemur (*Microcebus murinus*) (Goodman, 2003; Rahlfs & Fichtel, 2010), tamarins (*Sanguinus* spp.) (Isbell, 1994), Geoffroy's marmosets (*Callithrix geoffroyi*) (Searcy & Caine, 2003), and red‐nosed cuxiú (*Chiropotes albiniasus*) (Barnett et al., 2017). The latter species had a response similar to what we observed for *P. diadema*, in which predation attempts were accompanied by alarm calls, followed by silence and vigilance, and in some cases group dispersal (Barnett et al., 2017). The overall behavioral response of *P. diadema* is similar to the anti‐predator strategies of disrupting the predator, camouflaging, and strategic spatial positioning proposed for a congener, *P. edwardsi*, living in the rainforest of Ranomafana National Park, a species with a behavioral ecology very similar to *P. diadema* (Karpanty & Wright, 2007; Wright, 1998). A fosa‐specific anti‐predator strategy described for *P. edwardsi* comparable with our observations is splitting after the attack to reduce olfactory detection (Karpanty & Wright, 2007; Wright, 1998).

Changes in cohesion can reduce detectability, which may explain increased distance between individuals or the group splitting that we observed after attacks. Use of this strategy may relate to the sifaka locomotor style as a vertical clinger and leaper. Wright (1998) hypothesized that vertical clinging and leaping on tree trunks makes sifakas vulnerable to predation because they become easily detectable. However, Crompton and Sellers (2007) suggested that leaping from vertical supports can be an effective escape strategy because it allows a rapid and unpredictable change of directions. Leaping may also allow prey to escape across gaps; for example, red colobus increased leaping frequency in the presence of predators (Gebo et al., 1994).

Long‐distance, fast, asymmetrical leaping attacks can effectively disrupt a quadrupedal predator, such as a fosa, during the initial chase, but it is energetically suboptimal (Crompton & Sellers, 2007). Rather, the lemur response to fosa attacks is consistent with the hypothesis that a fosa cannot give chase for long or far: the sifakas (1) split up to confuse the predator and reduce the chance of being followed and (2) minimized their travel distance from the attack location and, after the first alerting in the “pursuit‐deterrent” response, opted for hiding in the canopy to reduce their detectability but remaining vigilant.

It is suggested that male sifakas tend to strategically position themselves ‐ below feeding females and juveniles and as the last ones traveling ‐ to protect the group against predators (Wright, 1998). This hypothesis would predict that males are attacked more frequently than females; however, we observed that fosas equally targeted males and females. This difference may be due to no or few young individuals in the groups in Betampona, which may change the protective behavior of males getting between predators and the rest of the group. Additionally, the small group sizes of just two adults (one male and female) can make group members vulnerable to predation independently of the sex of the individuals.

The Betampona population has higher average annual offtakes of individuals per group, whether considering confirmed fosa kills alone (0.42) or in combination with individuals that disappeared (1.04), compared to those for Ranomafana and Tsinjoarivo (with a maximum of 0.33) (Irwin et al., 2009). We also found a higher predation rate during this study compared to a year‐long study of diademed sifakas in Betampona in 2013 which reported the confirmed kill of one individual among three groups, corresponding to an average offtake of 0.33 individuals per year per group (Oliver, 2017). The higher rates of successful and attempted predation events that we observed more recently in Betampona could be attributed to a few factors: the fosa population may be larger in Betampona than 10 years ago or compared to other similarly‐sized forests, a similar number of fosas may be more active and successful at hunting sifakas in Betampona compared to other sites, or detection of predation by fosas in Betampona may be more common if the fossa are more habituated to human presence, the intensity of observation, or other conditions that exist at the field site. The fosas in Betampona, especially the ones ranging in the southwest area, might be partially habituated to human presence given the ongoing monitoring of the carnivoran and lemur populations and the frequent presence of researchers in the area (MFG annual report, 2019; Oliver, 2017; Rasambainarivo et al., 2018). Fosas in and around the Menabe and Kirindy Reserves have also been observed to become habituated to humans and venture into villages to hunt poultry more frequently compared to other sites (Merson et al., 2019). When surveyed, residents of villages immediately surrounding of the reserve reported that fosas ventured into the villages to hunt and kill free‐ranging chicken (MFG, unpublished data), which could also contribute to their habituation to human presence.

Compared to the fosa predation on sifaka in Betampona, there was only one case of death associated with an injury reported for a black‐and‐white ruffed lemur (*Varecia variegata*) in Betampona, during the same time period and using the same methods on the same number of focal groups as this study, which we cannot exclude as being caused by fosa predation (unpublished data, personal observation). In parallel, we found little evidence of fosa predation on both sifakas and black‐and‐white ruffed lemurs in Vohibe Forest over the same time period and studying the same number of groups using the same methods as this study. In Vohibe, we never directly observed a fosa while following either species, and there have been no fatalities for the black‐and‐white ruffed lemurs, but two infants of *P. diadema* were lost at that site from unknown causes (unpublished data, personal observation).

Fosas have densities of 0.26 individuals/km^2^ and home ranges of 920–2710 ha in dry forest (Hawkins & Racey, 2005; Wyza et al., 2020), and they have lower or similar densities (0.17–0.27) and bigger home ranges in the eastern rainforests (Gerber et al., 2010; Irwin et al., 2009; Murphy et al., 2018). However, a study of carnivorans in Betampona confirmed at least seven individual fosas, translating to a minimum density estimate of 0.31 individuals/km^2^ (Rasambainarivo et al., 2018). This estimate is higher than in other areas, and it is also likely a conservative estimate since the trapping area did not encompass the entire reserve (see Rasambainarivo et al., 2018), suggesting its actual density is even higher. Additionally, the entire size of the reserve (2228 ha) would only encompass one or two fosas' home ranges, based on the sizes reported for other areas, although fosas' home ranges can overlap (Wyza et al., 2020), especially for males (Hawkins & Racey, 2005). The total area over which the three diademed sifaka groups range would be encapsulated by one fosa home range, but the four observed attacks involved a minimum of two and maximum of four fosas, which suggests that there is increased hunting pressure on the sifaka population at Betampona. Female fosas are usually solitary except when caring for offspring, and males are also solitary but can be found in dyads of often related individuals, with one of the benefits reported for such associations being cooperative hunting (Gerber & Hawkins, 2022; Lührs et al., 2013). Most likely, the two fosas that jointly attacked BP3 were males, but we cannot exclude the possibility that they were an adult female and subadult offspring. Also, we cannot exclude that the non‐collared fosas observed in the first two attacks in June 2022 were the same individual and possibly one of the two fosas that participated in the February 2023 predation attempt.

All the fosa attacks and suspected and confirmed killings in this study happened in neighboring sifaka groups between February and September in two consecutive years, with 78% of reported cases occurring between April and September. The pattern in Betampona is consistent with the pattern of depredation in space and time in Ranomafana National Park (*P. edwardsi*) and Tsinjoarivo (*P. diadema*), where the deaths of lemurs in neighboring groups by predation from fosas occurred between January and September, but primarily in a 5‐month period (May–September), and was interspersed by years with no known cases of deaths by predation (Irwin et al., 2009). This timing coincides with the lean season for the sifakas (April–October) which could make them weaker, and there may be less availability of other prey for fosas during this cold season due to hibernation of other mammals (Irwin et al., 2009). Concentrated predation in time and space in the context of fragmented and isolated habitats is particularly relevant for conservation considerations. Although predators in continuous forests can spread their impact across different groups and prevent depletion of food resources, they are constrained in an isolated or fragmented landscape, and the concentration of kills can have consequences for small populations of endangered species (Irwin et al., 2009).

Dispersal and recolonization of empty niches are limited in small, isolated lemur populations such as the sifakas in Betampona, in which reproductive success and species survival are affected by compounding factors such as inbreeding and high infant mortality rates (MFG annual report, 2022), making this population particularly vulnerable to both demographic and environmental stochasticity such as weather‐related events (e.g., cyclones, El Niño Southern Oscillations, droughts) that can affect lemurs' reproduction and food availability (Dinsmore et al., 2021; Dunham et al., 2010; Wright, 2006). Groups in Betampona are small – all three groups were composed of two adult individuals at the time of the attacks – whereas in Ranomafana and Tsinjoarivo, the groups range from three to nine individuals, and at all three sites, one individual per group was killed during each predation event (Irwin et al., 2009). When groups are limited to two individuals, like in Betampona, not only are the risks of predation higher than in larger groups (Isbell, 1994), but the loss of one individual prevents any further reproduction (at least until a new individual immigrates in or the remaining individual joins a new group) and affects the whole population. Furthermore, if infants and juveniles are easier targets for predation, their death impacts the reproductive success and demography of the population and prevents the population from growing. In such a small population, the loss of reproductive‐age females is also worrisome, and in this study, we reported two: the female of BP3 who was preyed upon by a fosa and the female of BP10 who disappeared. The female of BP10 was the only known offspring that the female of group BP2 raised to maturity and dispersal, meaning that her disappearance also impacted the fitness of the female in BP2. Groups with more than one predation event disappeared both in Ranomafana and Tsinjoarivo because of emigration or killing by predation of the remaining individuals (Irwin et al., 2009), just like one of our focal groups was also extirpated because of fosa predation. These factors together present a risk to the local extinction of diademed sifakas.

Combined, the observations of successful and unsuccessful predation attempts, the disappearance of individuals from the study groups, and the discovery of the body of one of the focal individuals with evidence of fosa predation point to a heavy impact of predation on diademed sifakas in Betampona. As hypothesized for nonhuman primates, predation constitutes a cost both via the extreme cost of mortality and the ongoing cost of vigilance and other anti‐predator behaviors (Fichtel, 2012; Isbell, 1994; Stanford, 2002). High predation pressure is a proximate factor threatening the survival of endangered prey species, especially in environments with altered landscapes (Schneider, 2001), with significant conservation implications. When both predators and prey are threatened species, it is important to take their interactions into consideration in conservation planning (Canale & Bernardo, 2016). At the same time, monitoring anthropogenic pressure that can affect predator–prey dynamics, such as hunting from humans and dogs, also needs to be considered a high priority.

Our observations suggest that fosa predation impacts the survival and reproduction of diademed sifakas in Betampona. Examining fosa and lemur interactions in other areas of the reserve will indicate if there are particular areas of the site with higher predation pressure. Furthermore, additional studies are needed to understand if fosas are having the same impact on other species of lemurs within Betampona – particularly those that use lower parts of the canopy similar to the diademed sifakas. Given the compounding effects of habitat isolation and high hunting pressure, conservation management plans should incorporate predator–prey dynamics by assessing estimates of prey and predators' population densities over time as well as quantifying the incidence of predation pressure on prey populations. These data would provide information to guide conservation‐oriented decisions, such as translocation of predators and prey, or the creation of forest corridors as a strategy to allow mobility for predators and lemurs, reducing predator pressure and increasing gene flow for lemurs. Domestic animals (cats and dogs) can compete with natural predators or have a direct effect on prey populations. Habituating predators to the presence of human observers can also change predation intensity as it removes the fear of humans that may have protected lemurs from predation when humans are present. Conservation actions that consider ecological dynamics together with anthropogenic factors can help the long‐term viability of small and isolated populations while taking into consideration ecological community and interspecific dynamics.

## AUTHOR CONTRIBUTIONS

**G. Bonadonna:** Conceptualization (equal); data curation (lead); investigation (lead); methodology (equal); visualization (lead); writing – original draft (lead); writing – review and editing (lead). **O. M. Ramilijaona:** Conceptualization (equal); data curation (supporting); investigation (lead); methodology (supporting); visualization (supporting); writing – original draft (equal); writing – review and editing (supporting). **B. M. Raharivololona:** Conceptualization (equal); project administration (equal); supervision (equal); writing – review and editing (equal). **A. Andrianarimisa:** Conceptualization (equal); supervision (equal); writing – review and editing (equal). **H. Razafindraibe:** Conceptualization (equal); supervision (equal); writing – review and editing (equal). **K. Freeman:** Funding acquisition (equal); project administration (equal); resources (equal); writing – review and editing (equal). **F. Rasambainarivo:** Resources (equal); writing – review and editing (equal). **E. E. Wroblewski:** Conceptualization (lead); funding acquisition (lead); methodology (lead); project administration (lead); resources (lead); supervision (lead); visualization (equal); writing – original draft (equal); writing – review and editing (lead). **K. M. Milich:** Conceptualization (lead); funding acquisition (lead); methodology (lead); project administration (lead); resources (lead); supervision (lead); visualization (equal); writing – original draft (equal); writing – review and editing (lead).

## CONFLICT OF INTEREST STATEMENT

The authors declare that they have no conflict of interest.

## Supporting information


Data S1.

## Data Availability

All data supporting the results and scripts used to perform the analyses are available as supporting material.
