# Correction to “Response of diademed sifaka (Propithecus diadema) to fosa (Cryptoprocta ferox) predation in the Betampona Strict Nature Reserve, Madagascar”

**DOI:** 10.1002/ece3.11587

**Published:** 2024-06-17

Bonadonna G, Ramilijaona OM, Raharivololona BM, Andrianarimisa A, Razafindraibe H, Freeman K, Rasambainarivo F, Wroblewski EE, Milich KM. Response of diademed sifaka (*Propithecus diadema*) to fosa (*Cryptoprocta ferox*) predation in the Betampona Strict Nature Reserve, Madagascar. Ecol Evol. 2024 Apr 9;14(4):e11248. doi: 10.1002/ece3.11248. PMID: 38601854; PMCID: PMC11004663.

**Funding Information**: Eric P. and Evelyn E. Newman Charitable Foundation; Duke Lemur Center Field Improvement Research Grant; Living Earth Collaborative, Washington University in St. Louis, Saint Louis Zoo's Wildcare Institute.

**Acknowledgements**: This study is part of a larger project supported by the Living Earth Collaborative at Washington University in St. Louis through the Eric P. and Evelyn E. Newman Charitable Foundation and received additional financial support from the Duke Lemur Center Field Improvement Research Grant that paid for some fieldwork equipment and the Saint Louis Zoo's Wildcare Institute for additional personnel salary support. We thank the Saint Louis Zoo's Wildcare Institute and Missouri Botanical Garden for their input in the project development. We thank the Ministère de l'Environnement et du Développement Durable, the Direction Régionale de l'Environnement et du Dévloppement Durable and Madagascar National Parks for granting the research permits as well as for supporting Madagascar Fauna and Flora Group (MFG), Washington University in St. Louis, and University of Antananarivo's work in Betampona. We thank Leslie Paige for providing the satellite image of Betampona. Special thanks to Ingrid Porton (MFG Vice Chair of Research and Conservation) for providing valuable feedback and improving the manuscript. We thank MFG, in particular Jean Noel, and Missouri Botanical Garden's Fortunat Rakotoarivony who helped with field logistics. We thank the research assistants José Razanamalala and Maradona Alphons Tina for their dedication and assistance with data collection, and the MFG conservation agents Georges, T. Guy Celestin, Venance Rakoto, Doxy Razafindrabary, Maniriarison, Jose Sylvain, and N. Georgin who helped with data collection, Eugene and Paulette who cooked for us, and all the porters. We thank the editor and reviewers for their suggestions and comments that helped improve this manuscript's quality.

## Supporting information


Appendix S1

