# Supplementary material for: Correction to “Response of diademed sifaka (Propithecus diadema) to fosa (Cryptoprocta ferox) predation in the Betampona Strict Nature Reserve, Madagascar”
Source: Ecol Evol. 2024 Jun 17;14(6):e11587. doi: 10.1002/ece3.11587 (PMC11182775; doi:10.1002/ece3.11587)
Supplement: Supplementary file 1 — Appendix S1 [file ECE3-14-e11587-s001.docx]

**Response of diademed sifaka (*Propithecus diadema*) to fosa (*Cryptoprocta ferox*) predation in the Betampona Strict Nature Reserve, Madagascar - Appendix**

Bonadonna G., Ramilijaona O.M., Raharivololona B.M., Andrianarimisa A., Razafindraibe H., Freeman K., Rasambainarivo F., Wroblewski E.E., Milich K.M.

Table S1. Dataset with daily proportion of scans for each behavior calculated on the total number of scans per day, daily path length in meters (DPL m) and meters per observation hour (DPL m/hr) for each phase before (PRE) and after (T0, T1, T2) the four fosa attacks on the three diademed sifaka groups.

| PHASE | GROUP | DATE | scan count | F | NV | O | R | SG | SOC | T | V | DPL_m | obs h | DPL_m/h |
| --- | --- | --- | --- | --- | --- | --- | --- | --- | --- | --- | --- | --- | --- | --- |
| PRE | BP10 | 5/9/2022 | 117 | 0.17 | 0.23 | 0 | 0.23 | 0.03 | 0 | 0.15 | 0.19 | 1196 | 9.75 | 123 |
| PRE | BP10 | 5/10/2022 | 117 | 0.12 | 0.16 | 0 | 0.26 | 0.03 | 0 | 0.11 | 0.32 | 1044 | 9.75 | 107 |
| PRE | BP10 | 5/11/2022 | 88 | 0.26 | 0.17 | 0 | 0.14 | 0.02 | 0 | 0.25 | 0.16 | 788 | 7.33 | 107 |
| PRE | BP10 | 5/12/2022 | 116 | 0.16 | 0.25 | 0.01 | 0.15 | 0.02 | 0 | 0.26 | 0.16 | 1435 | 9.67 | 148 |
| PRE | BP10 | 5/13/2022 | 87 | 0.16 | 0.37 | 0 | 0.21 | 0.01 | 0 | 0.17 | 0.08 | 881 | 7.25 | 122 |
| PRE | BP10 | 6/7/2022 | 116 | 0.13 | 0.35 | 0.01 | 0.2 | 0 | 0.01 | 0.13 | 0.17 | 1193 | 9.67 | 123 |
| PRE | BP10 | 6/8/2022 | 56 | 0.3 | 0.04 | 0 | 0.27 | 0.05 | 0 | 0.18 | 0.16 | 631 | 4.67 | 135 |
| PRE | BP2 | 5/16/2022 | 42 | 0.12 | 0.17 | 0 | 0.17 | 0.02 | 0 | 0.29 | 0.24 | 344 | 3.5 | 98 |
| PRE | BP2 | 5/17/2022 | 13 | 0 | 0 | 0 | 0.92 | 0 | 0 | 0.08 | 0 | 15 | 1.08 | 14 |
| PRE | BP2 | 5/18/2022 | 114 | 0.19 | 0.25 | 0 | 0.25 | 0.03 | 0 | 0.23 | 0.06 | 1317 | 9.5 | 139 |
| PRE | BP2 | 5/19/2022 | 47 | 0.43 | 0.06 | 0 | 0.06 | 0 | 0 | 0.3 | 0.15 | 490 | 3.92 | 125 |
| PRE | BP2 | 5/31/2022 | 95 | 0.06 | 0 | 0 | 0.19 | 0.06 | 0 | 0.33 | 0.36 | 889 | 7.92 | 112 |
| PRE | BP2 | 6/1/2022 | 13 | 0.46 | 0 | 0 | 0.15 | 0.08 | 0 | 0.31 | 0 | 394 | 1.08 | 364 |
| PRE | BP3_1 | 2/6/2023 | 29 | 0.66 | 0.03 | 0 | 0 | 0.03 | 0 | 0.24 | 0.03 | 330 | 2.42 | 137 |
| PRE | BP3_1 | 2/7/2023 | 121 | 0.32 | 0.01 | 0 | 0.37 | 0.04 | 0.01 | 0.17 | 0.07 | 881 | 10.08 | 87 |
| PRE | BP3_1 | 2/8/2023 | 110 | 0.31 | 0.14 | 0 | 0.26 | 0.04 | 0 | 0.17 | 0.09 | 1218 | 9.17 | 133 |
| PRE | BP3_1 | 2/9/2023 | 120 | 0.28 | 0.11 | 0.03 | 0.17 | 0.04 | 0.04 | 0.23 | 0.1 | 1328 | 10 | 133 |
| PRE | BP3_1 | 2/27/2023 | 26 | 0.39 | 0 | 0 | 0.35 | 0.04 | 0 | 0.15 | 0.08 | 163 | 2.17 | 75 |
| PRE | BP3_2 | 7/11/2023 | 124 | 0.17 | 0.07 | 0 | 0.35 | 0.02 | 0 | 0.14 | 0.26 | 750 | 10.33 | 73 |
| PRE | BP3_2 | 7/12/2023 | 126 | 0.23 | 0.06 | 0 | 0.43 | 0.01 | 0 | 0.14 | 0.13 | 759 | 10.5 | 72 |
| PRE | BP3_2 | 7/13/2023 | 111 | 0.28 | 0.09 | 0.01 | 0.17 | 0.01 | 0 | 0.28 | 0.16 | 1292 | 9.25 | 140 |
| T0 | BP10 | 6/8/2022 | 53 | 0.02 | 0 | 0 | 0.19 | 0.02 | 0 | 0.02 | 0.76 | 172 | 4.42 | 39 |
| T0 | BP2 | 6/1/2022 | 88 | 0.16 | 0.22 | 0 | 0.19 | 0 | 0 | 0.07 | 0.36 | 157 | 7.33 | 21 |
| T0 | BP3_1 | 2/27/2023 | 6 | 0 | 0.83 | 0 | 0 | 0 | 0 | 0.17 | 0 | 73 | 0.5 | 146 |
| T0 | BP3_2 | 8/16/2023 | 32 | 0.09 | 0 | 0 | 0 | 0 | 0 | 0.19 | 0.72 | 376 | 2.67 | 141 |
| T1 | BP10 | 6/9/2022 | 80 | 0.2 | 0 | 0 | 0.19 | 0.01 | 0.03 | 0.28 | 0.3 | 1030 | 6.67 | 155 |
| T1 | BP10 | 6/10/2022 | 111 | 0.19 | 0.08 | 0 | 0.26 | 0.02 | 0.03 | 0.24 | 0.18 | 1173 | 9.25 | 127 |
| T1 | BP2 | 6/2/2022 | 107 | 0.21 | 0.79 | 0 | 0 | 0 | 0 | 0 | 0 | 160 | 8.92 | 18 |
| T1 | BP3_1 | 2/28/2023 | 127 | 0.37 | 0.03 | 0.02 | 0.07 | 0.09 | 0.06 | 0.18 | 0.18 | 1089 | 10.58 | 103 |
| T1 | BP3_1 | 3/1/2023 | 133 | 0.35 | 0.02 | 0 | 0.24 | 0.05 | 0.06 | 0.16 | 0.14 | 1129 | 11.08 | 102 |
| T1 | BP3_1 | 3/2/2023 | 130 | 0.34 | 0.02 | 0 | 0.28 | 0.01 | 0.03 | 0.19 | 0.14 | 1158 | 10.83 | 107 |
| T1 | BP3_1 | 3/3/2023 | 65 | 0.29 | 0.29 | 0 | 0.25 | 0 | 0.03 | 0.08 | 0.06 | 516 | 5.42 | 95 |
| T1 | BP3_2 | 8/17/2023 | 107 | 0.14 | 0.68 | 0 | 0.03 | 0 | 0 | 0.06 | 0.09 | 543 | 8.92 | 61 |
| T2 | BP10 | 6/27/2022 | 112 | 0.15 | 0.18 | 0 | 0.38 | 0.03 | 0.03 | 0.13 | 0.12 | 1161 | 9.33 | 124 |
| T2 | BP10 | 6/28/2022 | 99 | 0.19 | 0.27 | 0 | 0.28 | 0.01 | 0 | 0.1 | 0.14 | 1078 | 8.25 | 131 |
| T2 | BP10 | 6/30/2022 | 80 | 0.11 | 0.56 | 0 | 0.09 | 0 | 0 | 0.08 | 0.16 | 793 | 6.67 | 119 |
| T2 | BP10 | 7/1/2022 | 96 | 0.16 | 0.17 | 0.01 | 0.34 | 0.01 | 0.02 | 0.12 | 0.18 | 779 | 8 | 97 |
| T2 | BP2 | 6/20/2022 | 36 | 0.08 | 0.36 | 0 | 0.44 | 0 | 0 | 0.06 | 0.06 | 358 | 3 | 119 |
| T2 | BP2 | 6/21/2022 | 122 | 0.22 | 0.07 | 0 | 0.18 | 0.04 | 0.04 | 0.12 | 0.34 | 914 | 10.17 | 90 |
| T2 | BP2 | 6/22/2022 | 90 | 0.18 | 0.12 | 0.01 | 0.09 | 0.12 | 0.01 | 0.23 | 0.23 | 956 | 7.5 | 127 |
| T2 | BP2 | 6/23/2022 | 110 | 0.13 | 0.1 | 0 | 0.32 | 0.03 | 0.01 | 0.21 | 0.21 | 1230 | 9.17 | 134 |
| T2 | BP2 | 6/24/2022 | 69 | 0.15 | 0.06 | 0 | 0.54 | 0 | 0 | 0.13 | 0.13 | 335 | 5.75 | 58 |
| T2 | BP3_1 | 3/20/2023 | 35 | 0.34 | 0.17 | 0 | 0.03 | 0 | 0 | 0.31 | 0.14 | 569 | 2.92 | 195 |
| T2 | BP3_1 | 3/21/2023 | 124 | 0.24 | 0.13 | 0 | 0.31 | 0.01 | 0 | 0.21 | 0.11 | 1262 | 10.33 | 122 |
| T2 | BP3_1 | 3/22/2023 | 122 | 0.43 | 0.04 | 0.01 | 0.14 | 0.04 | 0.01 | 0.17 | 0.16 | 1272 | 10.17 | 125 |
| T2 | BP3_1 | 3/23/2023 | 123 | 0.42 | 0.07 | 0.01 | 0.11 | 0 | 0.02 | 0.25 | 0.13 | 1409 | 10.25 | 137 |
| T2 | BP3_1 | 3/24/2023 | 62 | 0.37 | 0.11 | 0 | 0.16 | 0.02 | 0 | 0.15 | 0.19 | 377 | 5.17 | 73 |
| T2 | BP3_2 | 9/5/2023 | 133 | 0.41 | 0.06 | 0 | 0.41 | 0.03 | 0 | 0.06 | 0.04 | 968 | 11.08 | 87 |
| T2 | BP3_2 | 9/6/2023 | 132 | 0.3 | 0.14 | 0 | 0.3 | 0.02 | 0 | 0.13 | 0.11 | 1355 | 11 | 123 |

Table S2. Results of post hoc Tukey’s test for pairwise comparisons of sequential phases before (PRE) and after the fosa attacks (T0, T1, T2). The test was performed on behaviors with ANOVA indicating significant variation amongst phases. Contrasts with p-value <0.05 are highlighted in bold.

| Behavior | contrast | estimate | SE | df | t-ratio | p-value |
| --- | --- | --- | --- | --- | --- | --- |
| Feeding | **PRE - t0** | **0.189** | **0.06** | **42** | **3.27** | **0.011** |
|  | PRE - t1 | 0.024 | 0.04 | 43 | 0.53 | 0.952 |
|  | PRE - t2 | 0.016 | 0.04 | 42 | 0.46 | 0.968 |
|  | t0 - t1 | -0.165 | 0.07 | 42 | -2.54 | 0.069 |
|  | **t0 - t2** | **-0.173** | **0.06** | **42** | **-2.92** | **0.028** |
|  | t1 - t2 | -0.007 | 0.05 | 42 | -0.16 | 0.999 |
| Vigilance | **PRE - t0** | **-0.320** | **0.07** | **43** | **-4.92** | **0.000** |
|  | PRE - t1 | -0.007 | 0.05 | 44 | -0.13 | 0.999 |
|  | PRE - t2 | -0.017 | 0.04 | 43 | -0.44 | 0.972 |
|  | **t0 - t1** | **0.314** | **0.07** | **43** | **4.28** | **0.001** |
|  | **t0 - t2** | **0.303** | **0.07** | **43** | **4.54** | **0.000** |
|  | t1 - t2 | -0.011 | 0.05 | 43 | -0.21 | 0.997 |

**Description of fosa attacks**

**BP2 -** On June 1^st^ 2022, the day of the fosa attack, the female was our focal. The fosa attack occurred in the northern part of the group’s home range (Fig 6A). The fosa was on a tree nearby the two sifakas, which were vigilant and emitting Zzuss-tsk alarm calls. After emitting a loud vocalization, the fosa attempted to catch the female in the canopy, but she escaped by moving rapidly away and staying at about 10m distance from the predator. The female remained stationary in the lower canopy and continued to be vigilant and emit Zzuss-tsk calls for 16 minutes (Fig. S1). After the unsuccessful attack, the fosa returned to the ground roaming in the area - we do not know the exact time the fosa left. After the attack, the female sifaka stayed in the same area for two and half hours and then traveled until she reached a big feeding tree where she stayed for the rest of the day (Fig. 6A).

**BP10 -** On 8 June 2022, the attack was directed towards the male although our focal individual for that day was the female. We first saw the male alarming and quickly moving through the canopy in the direction of the female as a fosa was climbing down from a tree near the male. Both sifakas immediately started a sequence of Zzuss-tsk vocalizations (Fig. S1). The fosa emitted a loud vocalization but it did not attempt a second attack. As the two sifakas left, the fosa climbed a tree and laid down on a branch in the lower canopy. It did not try to pursue the two sifakas as they fled. Both sifakas left the area while still emitting Zzuss-tsk alarm calls. The focal individual (female) spent the rest of the day of the attack being vigilant (Fig. 3) and we lost sight of the male.

**BP3 -** On 27 February 2023 (Case 1), the male was the focal individual although the female was present as well. At 5:00 PM, we saw two fosas on the ground, one of the two individuals was fitted with a radio collar. The fosa with the collar climbed on the tree where the two diademed sifakas were feeding meanwhile the other one stayed on the ground. Once the fosa reached the lower canopy, the two sifakas split by travelling in different directions while uttering the alarm call Zzuss-tsk. The fosa that was on the ground climbed the tree where the female was located, when she stopped vocalizing and moved away the fosa refocused its attention on the male that continued emitting Zzuss-tsk for three minutes (Fig. S1). At that point, the two fosas also stopped the chase. After 73m of displacement from the attack location, the male hid in the middle canopy, and was out of sight. On the day after the attack, we found the male alone east from where we left him the day before and not far away (44 m) from the attack location (Fig. 6C). The individual was vigilant and traveling while emitting the lost call Howl (Fig S1) until when the male and female rejoined at a place 50m away from the attack location.

On 8 August 2023, around 12:00 PM, an MFG agent performing routine data collection in the forest -within the area where group BP3 ranges - saw the pair of diademed sifakas being chased by a fosa. Other MFG agents joined the effort in following this case; they found the male alone at 2:40 PM and at 4:51 PM found the dead body of the female, which was half eaten. The MFG agent saw that the fosa returned to eat the remains of the female after he walked away from the body.

On 16 August 2023, eight days after the death of the female, we observed the lone male being the target of another predation attempt by a fosa (Case 2). The male came across a fosa sleeping on a tree trunk laying on the ground, the sifaka emitted Zzuss-Tsk vocalizations and then positioned himself in the middle canopy of a tree, staying vigilant (Fig. S1). After about 45 minutes the fosa returned below the tree where the male sifaka was and began to slowly climb the tree. After noticing that the fosa was very close to him, the lemur fled meanwhile emitting Zzuss-Tsk vocalizations (Fig. S1). The predator jumped immediately in an unsuccessful attempt to capture him. The location of this predation attempt was at 146 m from the fatal attack on the female that occurred the week before, and 120 m from the attack observed in February (Fig. 6D). We tried to follow the male sifaka and lost track of the fosa. We found the male at 2:10 PM on a large tree (DBH 60cm, height over 25m) at 52 m from the attack location.

The cases reported for BP2 have key differences. The first case involved two fosas attacking two individuals, and in the second case, a single male accidentally came across a fosa one week after the group was the target of a fosa predation event in which the female was killed. In both cases the sifakas hid in the canopy after the attacks, and they reduced their travel following an initial flight. Both attacks and the killing took place in the west and center-west area of the home range, and the individual(s) retreated toward the center and center-east area of the home range (Fig 6C and 6D).


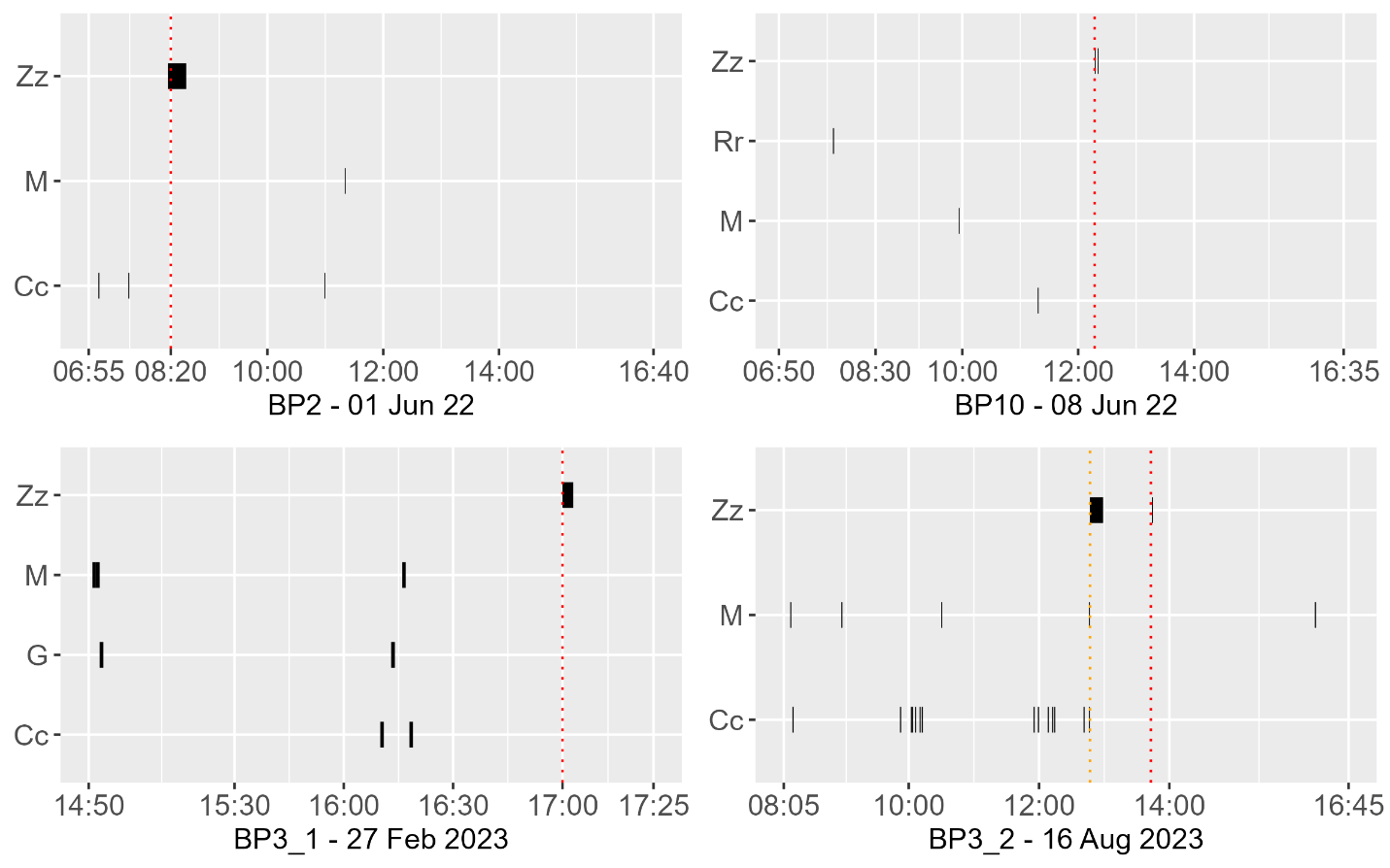


Fig S1. Time series with the behaviors recorded using all-occurrences sampling method on the day of each attack. Each plot represents the entire observation time for each day on each group (BP2, BP10, BP3 case 1 and 2). Red dotted lines indicate the time of the fosa attacks, the orange dotted line indicate when the male of BP3 came across the fosa. Zz: Zzuss/Zzuss-tsk; Rr: Roar; M: Marking; G: Allogrooming; Cc: Cohesion and contact calls (see manuscript for the description of vocalizations and reference therein).
